# Risk factors and long-term outcomes of oropharyngeal dysphagia in persons with multiple sclerosis: a systematic review protocol

**DOI:** 10.1186/s13643-024-02530-3

**Published:** 2024-05-02

**Authors:** Zahra Sadeghi, Mohamadreza Afshar, Asefeh Memarian, Heather L. Flowers

**Affiliations:** 1 Department of Speech Therapy, University of Social Welfare and Rehabilitation Sciences, Tehran, Iran; 2 Department of Speech Therapy, School of Rehabilitation, Tehran University of Medical Sciences, Tehran, Iran; 3 School of Rehabilitation Sciences, Faculty of Health Sciences, University of Ottawa, 200 Lees Avenue, Ottawa, ON K1S 5S9 Canada

**Keywords:** Predictors, Oropharyngeal dysphagia, Multiple sclerosis, Systematic review, Protocol

## Abstract

**Background:**

Oropharyngeal dysphagia (OPD) can be functionally debilitating in persons with multiple sclerosis (pwMS). OPD induces alterations in safety and efficiency of food and/or liquid ingestion and may incur negative sequalae such as aspiration pneumonia or malnutrition/dehydration. Early detection and timely management of OPD in pwMS could prevent such complications and reduce mortality rates. Identifying risk factors of OPD relative to its onset or repeat manifestation will enable the development of care pathways that target early assessment and sustained management. The aims of this systematic review are to compile, evaluate, and summarize the existing literature reporting potential risk factors and associated long-term outcomes (e.g., aspiration pneumonia, malnutrition, dehydration, and/or death) of OPD in pwMS.

**Methods:**

We will undertake a systematic review to identify studies that describe patterns and complications of OPD in pwMS. Variables of interest include predictors of OPD along with long-term outcomes. We will search MEDLINE, Embase, CINAHL, AMED, the Cochrane Library, Web of Science, and Scopus. We will consider studies for inclusion if they involve at least 30 adult participants with MS and report risk factors for OPD and/or its long-term outcomes. Studies will be excluded if they refer to esophageal or oropharyngeal dysphagia induced by causes other than multiple sclerosis. Study selection and data extraction will be performed by two independent assessors for abstract and full article review. We will present study characteristics in tables and document research findings for dysphagia-related risk factors or its complications via a narrative format or meta-analysis if warranted (e.g., mean difference and/or risk ratio measurements). All included studies will undergo risk-of-bias assessment conducted independently by two authors with consensus on quality ratings.

**Discussion:**

There is a lacune for systematic reviews involving risk factors and long-term outcomes of dysphagia in pwMS to date. Our systematic review will provide the means to develop accurate and efficient management protocols for careful monitoring and evaluation of dysphagia in pwMS. The results of this systematic review will be published in a peer-reviewed journal.

**Systematic review registration:**

PROSPERO CRD42022340625.

## Background

Oropharyngeal dysphagia (OPD) is common in multiple sclerosis (MS) [1] due to injury to the corticobulbar tracts, potentially involving the brainstem, the cerebellum [2, 3], and the cortex [4]. There may be a differing clinical course across types of MS, classified based on disease onset and progression over time [5]. The most frequent includes relapsing–remitting MS (RRMS), usually beginning with acute exacerbation and detrimental impacts that recover fully or partially over time. The other forms of MS are all defined as progressive, including secondary progressive MS (SPMS), primary progressive MS (PPMS), and progressive relapsing MS (PRMS). Patients with RRMS develop SPMS within 10 years of the onset of RRMS. PPMS is the least frequent type of MS and is characterized by deteriorating neurological function from disease onset coupled with a lack of remittance. Nevertheless, superimposed relapses are also evidenced in this type. Overall, the course of MS is unpredictable, and depending on the severity, the diversity of anatomic impacts, and the onset of associated lesions, its clinical manifestations are also heterogeneous.

Symptoms of OPD in MS may include coughing and/or choking on saliva or other liquid and food boluses, feelings of bolus sticking in the throat, the need to swallow multiple times per bolus, difficulty initiating a swallow (accompanied by drooling), and alterations to usual eating patterns (such as viscosity or texture changes) [3, 4, 6]. Oropharyngeal dysphagia may incur severe and multifaceted poor outcomes, such as aspiration pneumonia, malnutrition/dehydration [3, 4, 6], increased psychosocial comorbidities [7, 8], and even death during periods of medical instability [9]. Identifying risk factors for OPD in pwMS will provide the means to develop accurate and efficient management protocols for careful monitoring and evaluation by dysphagia experts. By extension, sustained management will permit timely and comprehensive care to mitigate potential serious complications.

In two recent systematic reviews, the authors provided an estimate of the pooled frequency of dysphagia in pwMS based on a range of evaluation methods, whether screening, clinical, or instrumental examination [1, 10]. Guan et al. [1] reported a pooled frequency estimate of 36% based on subjective screenings or cursory evaluations (such as the Dysphagia in Multiple Sclerosis Questionnaire, the water swallowing test, and various dysphagia checklists from individual clinical swallowing centers) and 81% based on objective measurements (such as videofluoroscopy or fiber-optic nasoendoscopy). More specifically, the frequency of dysphagia was 46% in pwMS when Expanded Disability Status Scale (EDSS) scores were stratified as 4.5 or higher and 40% for those below 3.0. Similarly, patients with longer disease duration (over 10 years) were more likely to have dysphagia symptoms compared with shorter disease duration.

Various individual studies have also shown a higher frequency of dysphagia with greater disability [11–15] and/or disease duration [15, 16]. Nevertheless, a few studies have reported that pwMS with low EDSS scores still had dysphagia [15, 17, 18]. To illustrate, Abraham et al. [18] reported that 43% of pwMS in their sample had dysphagia including 17% with low levels of disability (EDSS score lower than 2.5). Aghaz et al. [10] estimated the pooled frequency of dysphagia as 37% based on subjective evaluations or cursory checklists versus 47% for objective instrumental evaluations respectively. In contrast to the findings of Abraham et al. [18], they failed to demonstrate associations for the presence of dysphagia according to EDSS-based disease severity, duration of disease, or MS stage.

Taken together, reported frequencies of dysphagia hover around one-third of pwMS at a given point in time [1, 10], whereby varied frequencies relate primarily to evaluation methods, whether screening, clinical assessment, or instrumental evaluation. The most common patient-report tool used to identify dysphagia in pwMS is the Dysphagia in Multiple Sclerosis Questionnaire (DYMUS) [1, 10, 19], involving 10 items with very good reliability and internal consistency. However, frequencies of reported dysphagia based on questionnaires are lower than those based on standardized tools or instrumental evaluations [1]. In general, instrumental assessment remains the gold standard for dysphagia and aspiration detection, whether by videofluoroscopy or fiber-optic nasoendoscopy rather than various types of screening tools, bedside evaluations, or patient-reported questionnaires. Some pwMS may underestimate their dysphagia severity due to altered sensory appreciation of symptoms, despite instrumental evidence to the contrary.

In addition to our poor understanding of the frequency of dysphagia in pwMS, gaps exist regarding patterns of associations between disease severity, duration, or stage. Notwithstanding, certain predictive factors may well routinely accompany the expression of dysphagia in pwMS. Elucidating such information would require a comprehensive profile of patient groups with known disease severity, duration, and stage alongside MS type, neuroanatomical impacts, and concomitant deficits or disorders. For example, dysphagia may be precipitated by coexisting psychological or cognitive impairments [11, 18, 20]. Therefore, continual monitoring for risk of dysphagia in pwMS who also experience negative mental health symptoms or cognitive disorders [4, 20] is warranted. Furthermore, speech impairments (e.g., dysarthria) may provide good and readily identifiable clinical indicators for the presence of dysphagia in persons with neuromuscular diseases [21]. A systematic appraisal of the literature is required to identify the best available evidence for risk factors of dysphagia along with ensuing long-term sequelae in pwMS.

A systematic review constitutes the highest level of research evidence, especially if there is a quality evaluation and meta-analysis. Therefore, a comprehensive systematic review, aimed at establishing the predictors of dysphagia in pwMS, ideally identified with gold standard evaluation methods (such as instrumental assessment), could facilitate the development of new tools for screening or assessing dysphagia and inform practice guidelines. In addition, a close consideration of associated outcomes over the long term (e.g., pneumonia, poor social participation, death) could contribute to our understanding of prognostic indicators for particular patient groups. Consequently, our purpose is to search the existing literature to systematically identify the risk factors and associated outcomes of oropharyngeal dysphagia over the long term in persons with pwMS.

## Methods

The protocol of this systematic review has been registered in PROSPERO (registration number: CRD42022340625). We have applied PRISMA-P guidelines to develop this review protocol further. It served to direct our search strategy of databases and the gray literature as well as our data extraction and compilation methods. We will document our article selection results using the PRISMA flow diagram to delineate reasons for abstract and article exclusion until the final set of articles is identified. Our investigation of risk factors is in keeping with recommendations from the Cochrane Prognostic Methods Group (https://methods.cochrane.org/prognosis/) [22]. We are submitting the protocol prior to undertaking the full search or any subsequent processes such as abstract screening and full article evaluation.

### Operational definitions

Oropharyngeal dysphagia is defined as body and structure impairment [23] in swallowing physiology evidenced by expert clinical or instrumental assessment of function from the anterior aspect of the lips to inferior aspect of the upper esophageal sphincter. Diagnosis of multiple sclerosis is based on accepted criteria for both definite and probable MS, according to a classification scheme that involves expert clinical and objective evaluations (such as neuroimaging) [24].

### Data sources

We will conduct an electronic search in the following databases for abstracts in languages that the co-authors can read (English, French, German, Persian, Portuguese, Spanish, and Turkish). No publication date or study design restrictions will be imposed. Relevant databases will include MEDLINE, Embase, CINAHL, AMED, the Cochrane Library (CENTRAL), Web of Science, and Scopus. The MeSH and search terms used in the search strategy were developed a priori (Table 1). A research librarian will consult to enable valid adaptations of the MEDLINE terms into the other databases. Our MEDLNE search was conducted in OVID, revealing 189 citations (April 2023). We will also search international gray literature sources (e.g., OpenGrey and Dissertation Abstracts) and review the bibliographies and citations for all included articles in a reiterative manner until no further possible references are identified.

**Table 1:** MEDLINE search terms for the concepts dysphagia and multiple sclerosis

**Concepts**	**Entry**	**Keywords** (developed by A. M. and H. F.)
Dysphagia^a^	1	deglutition disorders/
	2	deglutition/
	3	enteral nutrition/
	4	((enteral or tube or gastric) adj feed$).ti,ab,kf
	5	((deglut$ or swallow$) adj3 (difficult$ or disorder$ or abnormal$ or delay$ or dysfunction$ or impair$ or problem$ or disabil$ or disabl$ or deficit$)).ti,ab,kf
	6	((deglut$ or swallow$) adj3 (behavior or behaviour or function$)).ti,ab,kf
	7	dysphag$.ti,ab,kf
	8	1 or 2 or 3 or 4 or 5 or 6 or 7
MS^b^	9	exp Multiple Sclerosis/
	10	Demyelinating diseases/
	11	(("multiple sclerosis" or MS) adj3 "chronic progress*").ti,ab,kf
	12	(("multiple sclerosis" or MS) adj3 "progress* relaps*").ti,ab,kf
	13	(("multiple sclerosis" or MS) adj3 "progress* chronic").ti,ab,kf
	14	(("multiple sclerosis" or MS) adj3 "relaps* progress*").ti,ab,kf
	15	(("multiple sclerosis" or MS) adj3 "secondary progress*").ti,ab,kf
	16	(("multiple sclerosis" or MS) adj3 "primary progress*").ti,ab,kf
	17	(("multiple sclerosis" or MS) adj3 "relaps* remi*").ti,ab,kf
	18	(("multiple sclerosis" or MS) adj3 "remi* relaps*").ti,ab,kf
	19	(("multiple sclerosis" or MS) adj3 "acute relaps*").ti,ab,kf
	20	(("multiple sclerosis" or MS) adj3 "optic* spinal").ti,ab,kf
	21	(("multiple sclerosis" or MS) adj3 "optic spinal").ti,ab,kf
	22	(("multiple sclerosis" or MS) adj3 "acute fulminat*").ti,ab,kf
	23	or\9–22
	24	8 and 23

*MS* multiple sclerosis

^a^Terms adopted or modified from Flowers et al. [25]

^b^Terms adopted or modified from Farinotti et al. [26]

### Eligibility criteria

Studies will be considered for inclusion if they have observational intent and involve retrospective or prospective consecutive or randomly selected sampling (either from a particular cohort or population). Study designs may include case series, cross-sectional, longitudinal, case–control, and/or other observational investigations as well as the control arm (i.e., participants who are not receiving trial-related interventions for MS or dysphagia) of randomized controlled trials. We will consider studies with at least 30 adults (18 years or older) with MS. Studies must include an aim to identify risk factors (e.g., MS subtype, disease duration, EDSS score, age, gender, smoking or alcohol use, psychological symptoms, cognitive impairments, and/or dysarthria) that may precipitate oropharyngeal dysphagia (OPD). We have chosen not to prespecify all possible risk factors as we seek to identify new potential risk factors. The body of evidence is small, and risk factors are likely underrepresented at present. Any potential new risk factors will provide a path for future researchers to investigate them in a comprehensive way and thus extend the literature and knowledge base in this respect.

Corresponding studies that include follow-up time points will contribute to our interest in long-term outcomes (e.g., detrimental medical, activity/participation, or quality-of-life outcomes). Ideally, such studies would have comparable follow-up periods (e.g., yearly) that span the course of the disease and document the outcomes relative to the absence/presence and/or severity classifications of OPD. However, we will not exclude any studies based on their follow-up points or overall time horizon.

During our review of abstracts and full articles, we will apply pre-defined exclusion criteria. That is, we will exclude studies involving convenience samples, those without extractable data (e.g., studies involving aggregate results for multiple etiologies rather than pwMS alone) for our outcomes of interest, and those reporting duplicate data. Any abstracts without corresponding full study publications will be excluded. We will also exclude articles without a clear sample of at least 30 pwMS and corresponding OPD (for at least a declared portion of the sample), identified by clinical or instrumental swallowing assessments. Finally, articles will be excluded if they do not conform to our operational definitions of OPD and MS or if they refer to oropharyngeal dysphagia induced by causes other than multiple sclerosis. We will contact authors when we cannot find full articles or when we wish to elucidate study characteristics such as method of dysphagia assessment. Our full systematic review reporting will conform to the Strengthening the Reporting of Observational Studies in Epidemiology (STROBE) checklist [27].

### Data collection

Study selection from primary articles will be performed in two stages:

I. Initial screening and coding of titles and abstracts whereby relevant abstracts (stage 1) will undergo full article review (stage 2) (Table 2)
II. Evaluation and coding of full articles for inclusion in the final sample (Table 2)

**Table 2:** Proposed hierarchical coding categories for abstract and full article review

Code category	Stage 1: Exclude if abstract	Stage 2: Exclude if full article
1	Is clearly a review, commentary, or opinion	Is clearly a review, commentary, or opinion
2	Clearly does not involve any adults (18 + years)	Clearly does not involve any adults (18 + years)
3	Clearly involves a sample size of less than 30 persons	Clearly involves a sample with fewer than 30 pwMS
4	Clearly has no participants with potential MS	Clearly has no participants with MS or MS diagnosis is outside operational definition (e.g., expert clinical diagnosis without instrumental or biomarker corroboration)
5	Clearly has no mention of dysphagia or terms relating to swallowing structures or physiology	Dysphagia identification is clearly outside operational definition (e.g., esophageal dysphagia or lacking clinical or instrumental assessment of swallowing function)
6	N/A	Outcomes relating to risk factors or long-term outcomes for OPD clearly absent or unextractable from dataset
7	Clearly involves same data as another abstract	Clearly involves same data as another article
ACCEPT	If no codes apply, accept for full review	If no codes apply, accept for inclusion

*OPD* oropharyngeal dysphagia, *pwMS* persons with multiple sclerosis

The review process will be conducted by two independent reviewers (blind to each other’s coding) across the two stages. Any discrepancies will be resolved by consensus discussion between the two reviewers, and, when agreement is not possible, a third reviewer (also a member of the research team) will read the abstract or article independently and contribute to a decision. All references for the excluded articles will be retained for documentation purposes.

One data extractor will identify pertinent information from the final set of included articles and compile it into a table or spreadsheet. Extracted data will be verified by a second independent reviewer to ensure the accuracy of information from the following categories:

i) Study characteristics: First author’s name, year of publication, country in which the study was conducted, study design, and size of the sample
ii) Study population and participant characteristics: Age, gender, MS type, disease duration, and EDSS score
iii) Diagnostic assessments for MS and dysphagia
iv) Risk factors for dysphagia whether related to MS (e.g., MS type, disease duration, and EDSS score), to patient characteristics (e.g., age, gender, and smoking or alcohol abuse), and/or to comorbidities (e.g., psychological symptoms, cognitive impairments, or dysarthria)
v) Follow-up assessments of dysphagia in terms of type and timing
vi) Frequency and impact (e.g., severity) of detrimental medical (e.g., aspiration pneumonia, dehydration, malnutrition, institutionalization, and mortality), activity/participation (e.g., fewer social engagements around meals), or quality-of-life outcomes.

### Risk of bias in individual studies

We will apply appropriate risk-of-bias evaluations [22, 28] such as the Newcastle–Ottawa scale (NOS) [29] as a quality evaluation of included obserervational studies. To illustrate, the NOS contains grading for categories of selection (e.g., sample representativeness), comparability (e.g., evaluation of confounders), and outcome (adequacy of follow-up period). Further, if warranted, the Cochrane Collaboration’s risk-of-bias tool will be used for randomized controlled trials, based on the domains: sequence generation, blinding of participants, blinding of outcome measurement, allocation sequence concealment, missing data, selective outcome reporting, and other biases such as sources of funding and conflicts of interest [30]. Additionally, the Quality in Prognosis Studies (QUIPS) tool will facilitate assessment for the risk factor studies [22]. For any type of quality appraisal (observational study quality scale, Cochrane’s risk-of-bias tool, or QUIPS), two authors will independently review the included studies and resolve discrepancies by discussion and consensus agreement within the review team.

### Data analysis

We will provide a descriptive synthesis of the findings from the included studies, structured around target population characteristics, type of assessments, and outcomes of interest. We will consider meta-analyses if there is an adequate number of studies and homogeneity of study populations and assessment methods. Otherwise, we will present a narrative synthesis of the results. We anticipate that there will be restricted scope for meta-analysis due to differing study populations and/or assessment methods along with a paucity of existing literature. Where studies have similar sample characteristics (including potential comparison groups), assessment methods, and corresponding outcomes, we will pool the results and apply various types of meta-analyses such as mean difference, standard mean difference, or Cox regression for continuous outcomes and risk ratio measurement or logistic regression for categorical outcomes depicted in forest plots along with their 95% confidence intervals (CIs). Finally, we will evaluate the overall strength of the evidence based on discussion among authors through application of a tool such as GRADE.

### Assessment of heterogeneity

If there is reason to consider meta-analysis, analyses will be performed using Cochrane’s Review Manager tool (Review Manager: RevMan [computer program]. The Cochrane Collaboration, 2024). We plan to assess study features such as participant age and sex, MS subtype, time course for follow-up, and primary outcome measures as the basis for determining if data pooling for meta-analysis is warranted. Subsequently, if meta-analysis is undertaken, we will apply and interpret the *I*^2^ statistic [30] as an indictor of heterogeneity relative to the number of studies and direction of effect using the following guide: mild (between 0 and < 25%), moderate (between 25 and < 50%), severe (between 50 and < 75%), and highly severe (between 75 and 100%). If there is moderate heterogeneity, we will present a supplementary qualitative synthesis of the findings.

### Analysis of subgroups or subsets

If sufficient data are available, subgroup analyses may be conducted for different OPD assessment methods (e.g., clinical bedside evaluation, fiber-optic nasoendoscopic evaluation, and/or videofluoroscopic evaluation) relative to MS type and risk factors. Similarly, long-term outcomes based on dysphagia status or severity levels will be analyzed according to follow-up periods or comparable overall time horizon.

### Assessment of publication bias

Publication bias will be evaluated using a funnel plot (i.e., plots of study results against precision) and Begg’s [31] and Egger’s [32] tests if an adequate number of studies (≥ 10) are identified. Additionally, we will incorporate Deek’s asymmetry test [33] to mitigate overestimation of effects when predictive modeling with odds ratios is applied for the determination of OPD across studies that involve low event proportions. However, if meta-analysis is not possible, publication bias will be assessed descriptively and involve documentation of direction of results across risk factors (whether significant or not) as well as potential follow-up time lags across studies.

## Discussion

Our search strategy is extensive compared to other recent systematic reviews in the field of multiple sclerosis [1, 34] given the inclusion of numerous sources and comprehensive search terms. We believe that it will yield a broad capture of abstracts internationally, but that many articles will derive from western or developed countries. This may be an important limitation because many underrepresented countries, such as Iran, have a high and increasing prevalence of pwMS in certain regions [35, 36].

Other systematic reviews have undertaken different lines of inquiry such as investigating the prevalence of dysphagia in pwMS without considering risk factors [1] or long-term outcomes [1, 10]. Thus, we will extend the knowledge base in a new content area (involving predictors and long-term outcomes of dysphagia in pwMS). Our identification of literature in the field of MS will provide new insights into the repercussions of dysphagia and offer direction for the development of screening protocols, assessment methods, and improved therapeutic management in pwMS. In the event that our findings elucidate multiple predictors (e.g., related to MS, patient characteristics, and/or comorbidities) and varied outcomes (e.g., medical, activity/participation, or quality of life), they may warrant publication in multiple peer-reviewed papers.

We anticipate that various limitations will result during our search of the literature. First, studies may not report the timeframe between dysphagia onset, assessments, and associated outcomes. Second, dysphagia identification in specific studies might be based on cursory screenings, patient self-report (and potentially non-standardized) questionnaires, and/or subjective clinical assessments rather than on instrumental reference tests such as videofluoroscopy and fiber-optic nasoendoscopy. Finally, it may be difficult to pool results from the existing literature for some of the risk factors or outcomes if investigations restrict enrolment to particular types of pwMS, involving subsamples of larger studies, or if they fail to incorporate shared definitions and research methods in the field of multiple sclerosis [36].

## Conclusion

Although the frequency of dysphagia in pwMS has been a topic of inquiry within the past two decades [1, 10], a poor understanding of associations to disease-related risk factors and negative outcomes remains. Our proposed systematic review will address such a gap in the literature, as we will attempt to elucidate risk factors of dysphagia and long-term outcomes from observational studies reporting frequencies of dysphagia over the long term. Where relevant, we will pool results across studies or extract individual-level data that may permit us to model risk factors of dysphagia and/or its associated long-term outcomes in pwMS. Our inquiry will offer the means to inform best practices in the early detection of dysphagia and provide information that can be incorporated into guidelines and clinical practice initiatives for the management of dysphagia in pwMS.

## Data Availability

Not applicable.

## Abbreviations

OPD: Oropharyngeal dysphagia
pwMS: Persons with multiple sclerosis
MS: Multiple sclerosis
EDSS: Expanded Disability Status Scale
DYMUS: Dysphagia in Multiple Sclerosis Questionnaire
STROBE: Strengthening the Reporting of Observational Studies in Epidemiology
NOS: Newcastle-Ottawa scale

## References

[1] Guan X-L, Wang H, Huang H-S, Meng L (2015). Prevalence of dysphagia in multiple sclerosis: a systematic review and meta-analysis. Neurol Sci.

[2] Tassorelli C, Bergamaschi R, Buscone S, Bartolo M, Furnari A, Crivelli P (2008). Dysphagia in multiple sclerosis: from pathogenesis to diagnosis. Neurol Sci.

[3] Marchese-Ragona R, Restivo D, Marioni G, Ottaviano G, Masiero S, Staffieri A (2006). Evaluation of swallowing disorders in multiple sclerosis. Neurol Sci.

[4] Calcagno P, Ruoppolo G, Grasso M, De Vincentiis M, Paolucci S (2002). Dysphagia in multiple sclerosis–prevalence and prognostic factors. Acta Neurol Scand.

[5] Katz Sand IB, Lublin FD. Diagnosis and differential diagnosis of multiple sclerosis. Continuum. 2013;19(4 Multiple Sclerosis):922-43. 10.1212/01.CON.0000433290.15468.21. 10.1212/01.CON.0000433290.15468.21 23917094

[6] Prosiegel M, Schelling A, Wagner-Sonntag E (2004). Dysphagia and multiple sclerosis. Int MS J.

[7] Ekberg O, Hamdy S, Woisard V, Wuttge-Hannig A, Ortega P (2002). Social and psychological burden of dysphagia: its impact on diagnosis and treatment. Dysphagia.

[8] Verdonschot RJ, Baijens LW, Vanbelle S, van de Kolk I, Kremer B, Leue C (2017). Affective symptoms in patients with oropharyngeal dysphagia: a systematic review. J Psychosom Res.

[9] Sadovnick A, Eisen K, Ebers GC, Paty DW (1991). Cause of death in patients attending multiple sclerosis clinics. Neurology.

[10] Aghaz A, Alidad A, Hemmati E, Jadidi H, Ghelichi L (2018). Prevalence of dysphagia in multiple sclerosis and its related factors: systematic review and meta-analysis. Iran J Neurol.

[11] Thomas FJ, Wiles C (1999). Dysphagia and nutritional status in multiple sclerosis. J Neurol.

[12] Hartelius L, Svensson P (1994). Speech and swallowing symptoms associated with Parkinson’s disease and multiple sclerosis: a survey. Folia Phoniatr Logop.

[13] De Pauw A, Dejaeger E, D’hooghe B, Carton H (2002). Dysphagia in multiple sclerosis. Clin Neurol Neurosurg.

[14] Herrera W, Zeligman BE, Gruber J, Jones MC, Pautler R, Wriston R (1990). Dysphagia in multiple sclerosis: clinical and videofluoroscopic correlations. J Neurol Rehabil.

[15] Sadeghi Z, Afshar M, Ebadi A, Baghban K, Qureshi ZS (2020). Swallowing disorder in multiple sclerosis: modified version of the screening tool. Arch Rehabil.

[16] Tarameshlu M, Azimi AR, Ghelichi L, Ansari NN (2017). Prevalence and predictors of dysphagia in Iranian patients with multiple sclerosis. Med J Islam Repub Iran.

[17] Pajouh SD, Moradi N, Yazdi MJS, Latifi SM, Mehravar M, Majdinasab N (2017). Diagnostic evaluation of dysphagia in multiple sclerosis patients using a Persian version of DYMUS questionnaire. Mult Scler Relat Disord.

[18] Abraham S, Scheinberg LC, Smith CR, LaRocca NG (1997). Neurologic impairment and disability status in outpatients with multiple sclerosis reporting dysphagia symptomatology. J Neurol Rehabil.

[19] Bergamaschi R, Crivelli P, Rezzani C, Patti F, Solaro C, Rossi P (2008). The DYMUS questionnaire for the assessment of dysphagia in multiple sclerosis. J Neurol Sci.

[20] Sadeghi Z, Ghoreishi ZS, Flowers H. et al. Depression, anxiety, and stress relative to swallowing impairment in persons with multiple sclerosis. Dysphagia. 2021;36:902–9. 10.1007/s00455-020-10207-x. 10.1007/s00455-020-10207-x 33783621

[21] Wang BJ, Carter FL, Altman KW. Relationship between dysarthria and oral-oropharyngeal dysphagia: the present evidence. Ear Nose Throat J. 2020:145561320951647. 10.1177/0145561320951647. 10.1177/0145561320951647 33044841

[22] Riley RD, Ridley G, Williams K, Altman DG, Hayden J, de Vet HC. Prognosis research: toward evidence-based results and a Cochrane methods group. J Clin Epidemiol. 2007;60(8):863-5. 10.1016/j.jclinepi.2007.02.004 17606185

[23] World Health Organization. International classification of functioning, disability and health: ICF. World Health Organization; 2001. https://iris.who.int/handle/10665/42407.

[24] Poser CM, Paty DW, Scheinberg L, McDonald WI, Davis FA, Ebers GC (1983). New diagnostic criteria for multiple sclerosis: guidelines for research protocols. Ann Neurol.

[25] Flowers H, Bérubé D, Ebrahimipour M, Perrier M-F, Moloci S, Skoretz S (2019). Swallowing behaviours and feeding environment in relation to communication development from early infancy to 6 years of age: a scoping review protocol. BMJ Open.

[26] Farinotti M, Vacchi L, Simi S, Di Pietrantonj C, Brait L, Filippini G (2012). Dietary interventions for multiple sclerosis. Cochrane Database Syst Rev.

[27] Von Elm E, Altman DG, Egger M, Pocock SJ, Gøtzsche PC, Vandenbroucke JP (2007). The Strengthening the Reporting of Observational Studies in Epidemiology (STROBE) statement: guidelines for reporting observational studies. Lancet.

[28] Ma L-L, Wang Y-Y, Yang Z-H, Huang D, Weng H, Zeng X-T (2020). Methodological quality (risk of bias) assessment tools for primary and secondary medical studies: what are they and which is better?. Mil Med Res.

[29] Wells G, Shea B, O’Connell D, et al. The Newcastle-Ottawa Scale (NOS) for Assessing the Quality of Nonrandomised Studies in Meta-Analyses. 2011. http://www.ohri.ca/programs/clinical_epidemiology/oxford.asp.

[30] Higgins JP, Altman DG, Gøtzsche PC, Jüni P, Moher D, Oxman AD (2011). The Cochrane Collaboration’s tool for assessing risk of bias in randomised trials. BMJ.

[31] Begg CB, Mazumdar M (1994). Operating characteristics of a rank correlation test for publication bias. Biometrics.

[32] Egger M, Davey Smith G, Schneider M (1997). Bias in meta-analysis detected by a simple, graphical test. BMJ.

[33] Deeks JJ, Macaskill P, Irwig L (2005). The performance of tests of publication bias and other sample size effects in systematic reviews of diagnostic test accuracy was assessed. J Clin Epidemiol.

[34] Mirmosayyeb O, Ebrahimi N, Shekarian A, Afshari-Safavi A, Shaygannejad V, Barzegar M, Bagherieh S (2023). Prevalence of dysphagia in patients with multiple sclerosis: a systematic review and meta-analysis. J Clin Neurosci.

[35] Bagherieh S, Mirmosayyeb O, Vaheb S, Afshari-Safavi A, Barzegar M, Ashtari F, Shaygannejad V (2023). Prevalence and incidence of multiple sclerosis in Isfahan Province, Iran: a 25-year population-based study. Mult Scler Relat Disord.

[36] Cohen JA, Trojano M, Mowry EM, Uitdehaag BM, Reingold SC, Marrie RA (2020). Leveraging real-world data to investigate multiple sclerosis disease behavior, prognosis, and treatment. Mult Scler J.

